# Identity dimension of rural women and the sexual and reproductive health

**DOI:** 10.1590/0034-7167-2022-0298

**Published:** 2023-08-21

**Authors:** Michelle Barbosa Moratório de Paula, Ana Beatriz Azevedo Queiroz, Ívis Emília de Oliveira Souza, Anna Maria de Oliveira Salimena, Helen Petean Parmejiani, Ana Luiza de Oliveira Carvalho

**Affiliations:** I Universidade Federal do Rio de Janeiro. Rio de Janeiro, Rio de Janeiro, Brazil.; II Universidade Federal de Juiz de Fora. Juiz de Fora, Minas Gerais, Brazil.; III Fundação Universidade Federal de Rondônia. Porto Velho, Rondônia, Brazil.

**Keywords:** Rural Population, Women, Sexual Health, Reproductive Health, Psychology Social, Población Rural, Mujeres, Salud Sexual, Salud Reproductiva, Psicología Social, População Rural, Mulheres, Saúde Sexual, Saúde Reprodutiva, Psicologia Social

## Abstract

**Objectives:**

to analyze the social representations of rural women about being a woman in the rural context and its implications for sexual and reproductive health.

**Methods:**

this is a descriptive qualitative study with data triangulation, based on the Theory of Social Representations, developed with 31 women who live in the rural context of Minas Gerais (MG/BR). An in-depth interview with a semi-structured script was conducted. A lexical analysis was performed with the help of the ALCESTE 2012 software.

**Results:**

the family relationship, especially the couple’s, demonstrated subjectivities and was permeated by violence and normalized sexual practice. The imagery dimension of the ideal family seems to be responsible for exerting domination over rural women.

**Final Considerations:**

rural women are subject to the norms and prescriptions of a patriarchal society. It is urgent to increase attention to sexual and reproductive health in an egalitarian and liberating way in order to minimize the consequences of machismo and conservatism.

## RESUMO

**Objetivos:**

analisar as representações sociais de mulheres rurais sobre ser mulher no contexto rural e suas implicações na saúde sexual e saúde reprodutiva.

**Métodos:**

trata-se de um estudo descritivo, com triangulação dos dados, abordagem qualitativa, fundamentado na Teoria das Representações Sociais, realizado com 31 mulheres que vivem no contexto rural em Minas Gerais (MG/BR). Realizou-se entrevista em profundidade com roteiro semiestruturado. A análise foi do tipo lexical com auxílio do software Alceste 2012.

**Resultados:**

foram evidenciadas as subjetividades do relacionamento familiar, em especial do casal, sendo permeado pela violência e pela prática sexual normatizada. A dimensão imagética da família ideal parece ser responsável por exercer a dominação da mulher rural.

**Considerações Finais:**

a mulher rural encontra-se submetida às normas e prescrições de uma sociedade patriarcal. Faz-se urgente ampliar a atenção sobre a saúde sexual e saúde reprodutiva de forma igualitária e libertadora, no sentido de minimizar as consequências do machismo e do conservadorismo.

## RESUMEN

**Objetivos:**

analizar las representaciones sociales de las mujeres rurales sobre ser mujer en el contexto rural y sus implicaciones para la salud sexual y reproductiva.

**Métodos:**

estudio cualitativo descriptivo con triangulación de datos, basado en la Teoría de las Representaciones Sociales, desarrollado con 31 mujeres que viven en el contexto rural de Minas Gerais (MG/BR). Se realizó una entrevista en profundidad con guión semiestructurado. Se realizó un análisis léxico con la ayuda del software ALCESTE 2012.

**Resultados:**

la relación familiar, especialmente de pareja, demostró subjetividades y estuvo permeada por la violencia y la práctica sexual normalizada. La dimensión imaginaria de la familia ideal parece ser la responsable de ejercer la dominación sobre las mujeres rurales.

**Consideraciones Finales:**

la mujer rural está sujeta a las normas y prescripciones de una sociedad patriarcal. Es urgente aumentar la atención a la salud sexual y reproductiva de manera igualitaria y liberadora para minimizar las consecuencias del machismo y el conservadurismo.

## INTRODUCTION

Between the 1960s and 1980s, with the accelerated Brazilian urbanization, there were significant changes in the structure of occupation and jobs, with transformations in terms of income generation, lifestyle and population health. These transformations also brought different definitions of rural and urban areas^(1)^. The Brazilian rural area is no longer restricted to activities related to agriculture and agribusiness. In recent decades, it has gained new functions, classified as agricultural and non-agricultural, being identified as a space for food production, foreign exchange generation and deposit of potential labor for urban and industrial development^(2)^.

The rural population has suffered a process of rarefaction all over the world, even though such a population is still quite expressive in Brazil and one of the largest contingents in Latin America^(3)^. The reduction in the rural population may be related to inequalities characterizing this context, such as distance from large centers, lack of employment, reduced access to information, education, security and health services^(4, 5)^.

According to the last census carried out by the Brazilian Institute of Geography and Statistics (Portuguese acronym: IBGE) in 2010, the rural population represents 15.3% in Brazil, 48% of which are female^(6)^. This significant number presents specificities in relation to sexual and reproductive health (SRH). Among the various particular factors that influence this female segment, the strong connection with nature, gender inequalities, greater poverty, low education, as well as difficulties in accessibility and access to health services stand out^(7)^.

In 2011, the National Policy for the Comprehensive Health of Rural and Forest Populations was published through Ordinance No. 2,866, which was revoked, but maintained by a new Ordinance of the Ministry of Health of Consolidation No. 1 of 28/02/2017, aimed at reducing vulnerabilities through comprehensive actions for women’s health, considering the SRH^(8)^.

Faced with this context, different international and national studies^(9,10,11,12)^ have pointed to the compromise of women’s health imposed by the experience of the rural context due to gender issues, such as interpersonal and sexual violence, social inequalities, unwanted pregnancies and sexually transmitted infections (STIs). Given this scenario of vulnerabilities, the debate on women’s health takes on special contours when it comes to the rural context.

These characteristics outline the problem and complexity sustaining the theme studied, as being a woman in the rural context and taking care of the SRH is associated with social, political, economic, contextual, gender and behavioral issues. In this perspective, the identity aspects of rural women that govern their thoughts and attitudes towards their SRH are linked to the location where they live and in particular, to social issues extrapolating organic, physical, natural or objective elements.

From the above, it is questioned how lifestyle, social relations, culture and everyday life are related in the process of elaborating social representations regarding the identity dimension of being a woman in the rural context and the behaviors and attitudes that impact their SRH. The thoughts and decision-making of these women concerning their SRH are not reduced to the biological aspect, and include their subjectivities, cultural values, beliefs and traditions.

The premise of the social representations theory (SRT) is that the conformation of human thought takes place based on the various group insertions and in different fields such as social, historical, political and cultural^(13)^. Individual members in a social space end up producing a sense of belonging, which defines their own identity and way of being in the world, bringing to light singularities such as individuals, groups and social subjects^(14)^. Therefore, it is important to understand how women inserted in rural areas build their meanings as women, especially within a social context where gender relations are strongly unequal and hierarchical, legitimizing behaviors of domination and subordination^(9)^.

The social representation (SR) is a practical knowledge that guides behaviors, attitudes and practices expressed in a symbolic way in the cultural vision and within specific groups. Given this context, the construction of the social fabric in which rural women are immersed makes it possible to reach subjectivities, knowledge and practices that are related to their SRH and impact the quality of their lives.

## OBJECTIVES

To analyze the social representations of rural women about being a woman in the rural context and its implications for the sexual and reproductive health.

## METHODS

### Ethical Aspects

The research complied with CNS Resolution No. 466/2012 and obtained institutional authorization from the Municipal Health Department of Santa Rita de Ibitipoca (MG/BR). The project was approved by the Research Ethics Committee of the Escola de Enfermagem Anna Nery/São Francisco de Assis School Hospital. Participants were identified with an alphanumeric code composed of the interviewee’s initials, followed by the order number of interviews (Int. 01, Int. 02...).

### Type of Study

Descriptive qualitative study in which the SRT was applied in the procedural aspect as a theoretical and methodological contribution to understand the process of elaboration and the content of social representations about being a woman in the rural context.

The SRs have a conventional and prescriptive nature that models objects, people or events according to the language, time and culture of a group^(15)^. Therefore, subjects see reality through conventions, traditions and determined social models^(14)^. This theory allows understanding thoughts and actions that are mobilized by affections before vulnerability scenarios, especially in the area of the SRH^(16)^.

In the context of the present study, traditions and social models are norms that end up governing the adoption of attitudes, the construction of processes of formation of conducts and orientation of communications of rural women within their social context.

### Field of Study

The study was conducted in the municipality of Santa Rita de Ibitipoca, located in the Zona da Mata Mineira, state of Minas Gerais (BR). According to the last census, the municipality has a population of 3,583 inhabitants, of which 38% live in rural areas and 45.5% are women^(17)^.

### Participants

Participants were 31 women who live in the rural area of the municipality. The end of data production was based on a preliminary analysis, when sufficient data were obtained to understand the phenomenon. According to the scientific literature for qualitative research on SRT, this saturation process usually occurs at between 20 and 30 interviews^(18)^.

Inclusion criteria adopted were women over 18 years old, registered in the Family Health Strategy (FHS) of the municipality, who have always lived in rural areas. Migration to urban areas and the return to rural areas can contribute to different representations from those of people who lived exclusively in rural areas, as they can lead to thoughts, attitudes and behaviors of urban centers. Women with any cognitive deficit diagnosed by the physician and recorded in the medical record were excluded.

### Data Collection, Organization and Analysis

This field of study was chosen because one of the researchers worked for more than six years as a nurse in the FHS in this scenario. The daily care of these women made it possible to observe the studied phenomenon. The selection of participants was based on convenience, mediated by community health agents, who previously indicated women that could be potential participants.

The study was explained at the initial contact with these women. Later, if there was interest in participating, a meeting was arranged for data collection. The interviews took place at the participant’s home or at support health centers in rural areas, according to the woman’s preference.

After signing the informed consent, a questionnaire was applied to obtain the socioeconomic and demographic profile and data related to the SRH with the purpose of characterizing participants. Information on schooling, religion, marital status, occupation, personal and family income, active sex life, contraceptives, children, obstetric history and sexually transmitted infections was gathered.

Then, the in-depth individual interview technique was used. It included a script with the following open-ended and semi-directive questions: How is your partner with you? How does he treat you? And your children? Does your partner help you in raising children? How? What do you think about women who are beaten by their partners? What about your sex life? Tell me a little about it. Do you think your sex life influences your everyday life? How has it influenced?

The interviews took place from February 2017 to August 2018, and lasted an average of one hour. Data were recorded on a digital device and later transcribed in full. Data analysis was performed with support of the *Analyse Lexical par Contexte d’un Ensemble de Segments de Texte* (ALCESTE) software, 2012 version.

The ALCESTE software was used to facilitate data exploration, highlighting the lexicons conveyed by participants and providing information about the rationality of their speeches. The software captures groups and performs lexical classifications, while researchers track the meanings of the formed classes, name them, and perform the content analysis based on the distribution and association of lexicons^(19)^. Thematic block 3 analyzed in this study deals with the multidimensionality of the identity aspects of rural women and their impact on the SRH.

Lexical analysis was carried out using the descending hierarchical classification (DHC), the ascending hierarchical classification (AHC) and the elementary context units (ECUs). The DHC is presented through a table with reduced forms of words and all the lexicons related to words with higher statistical association (Phi)^(20)^. The AHC is displayed through a growing classification tree, in which it is possible to observe the interconnections between lexicons of each class. Finally, the ECUs allow the understanding of lexical classes formed by ALCESTE based on Phi.

The DHC, AHC and ECU results were analyzed and interpreted in the light of the theoretical framework adopted in the study, configuring data triangulation, which is essential for deepening and validating the results, enabling a multidimensional understanding of the studied phenomenon^(21)^.

## RESULTS

The socioeconomic and demographic profile of participants shows that this is a segment of women aged between 20 and 74 years old, mostly Catholic, married, with a low level of education and domestic work without personal income as the main occupation. Only a small number of participants consisted of young people with higher schooling and insertion in the labor market. Although households have electricity, basic sanitation is lacking. The health service used was exclusively the public, and the main source of information was television.

Regarding characteristics of the SRH, participants were multiparous, with home births accompanied by midwives. In the vast majority, the first sexual intercourse happened after the age of 20 years in the marriage, and they had an active sex life without desire at a frequency of once–twice a week without the use of contraceptive methods.

The analysis of the ALCESTE program was based on 3,478 different words, with 3,6917 occurrences and 75% utilization. The discursive material was divided into three large groups: the first thematic block included classes 5, 7 and 2; the second thematic block, classes 4, 6; and the third, classes 1, 8, 3.

The first thematic block brings the daily life of rural women in the domestic environment to which the vast majority are restricted, detecting a triple work shift. In the second block, the practical dimension in relation to the SRH emerges, when they portray reproductive planning, gynecological-obstetric care and the imagery dimension of motherhood as a divinity and inexorable destiny of rural women. Finally, block 3, which regards the objective of this study, shows subjectivities of the family relationship, especially of the couple, permeated by violence, a normalized sexual practice and the division of care for the child, establishing the mother as the caretaker and the father as the punitive educator. It also brings the imagery dimension of the ideal family, which seems to be responsible for exercising the domination of rural women. In this sense, this block was called “The family as an identity element of rural women”.

### Class 1 – Motherhood and fatherhood as elements of belonging in the rural context

**Figure 1:**
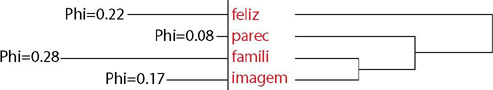
Ascending hierarchical classification of class 1 (happy, seems, family, image)

Class 1 was formed by 121 ECU, represented by 20% of the material, the largest in representation, with 83 analyzable words. It portrays the function of identity and belonging of rural women and men, which seems to be structured within the traditional family context. Women bring an imagery dimension of an ideal family formed by mother, father and children, and only in this family context do women achieve happiness. The lexicons *family* (phi=0.28), *happy* (phi=0.22), *image* (phi=0.17) and *seems* (phi=0.08) are interconnected in the AHC (Figure 1).

*Family is everything for me, father, mother and children! Money is nothing, what we need to be happy is to have a family.* (*Int_31)

The identity image of the ideal nuclear family carries a socially pre-established role for each of its members. The interconnections between semantic forms regarding the father’s identity function arise in this context; *education* (phi= 0.28), *dad* (phi= 0.23), *children* (phi= 0.33), *raising* (phi= 0.27), *help* (phi=0.24). Those of the mother are *mom* (phi= 0.12), *affection* (phi= 0.23), *attention* (phi= 0.17). Below are some ECU that demonstrate this result:

*My husband more or less helped me in raising my children, only in terms of education, punishing them and telling them off, but not watching the boys. I take care of that part. That’s my role.* (*Int_30)

*He helps me in the education of our children, they have enormous respect for their dad, when they do something wrong, they ask me not to tell the father, but I do tell, so they won’t do any more wrong things. They cry, but I tell him so he can give an education, but taking care and giving attention is with me.* (*Int_31)

Still with regard to the group to which men and women belong, in some ECU, a portion of rural women judge paternal identity as null, with women having to assume maternal and paternal roles alone.

*My husband helped little in the upbringing, so much so that children don’t care about him. They say I’m mom and dad. He knows how to give affection; I raised and took care of them alone.* (*Int_21)

*I guess what people don’t receive, they don’t know how to give. In his family, there were 15 children. He had no affection, attention. I do everything by myself, he’s never helped at all.* (*Int_18)

Class 8

### The identity of rural women in the family context: naturalized and justified violence

Class 8 gathers 46 ECU, represented by 8% of the material and 90 analyzable words. This class brings issues related to the understanding of being a rural woman in the family context where these women build their identity and experience family and domestic violence, which is naturalized and justified. The more associated lexicons with this class are *going to* (phi= 0.36), *arrive* (phi= 0.28), *drinking* (phi= 0.28), *argue* (phi= 0.28), *street* (phi= 0 .22), *son* (phi= 0.22). These words are associated with the daily life of rural women, permeated by domestic violence and magnified by alcohol abuse.

**Figure 2:**
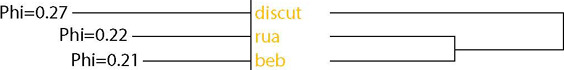
Ascending hierarchical classification of class 8 (argue, street, drinking)

The interconnection between lexicons *argue* (phi= 0.27), *street* (phi= 0.22), *drink* (phi=0.21), as identified in the AHC below (Figure 2), demonstrates that the male practice of consuming alcoholic beverages occurs on the street, that is, in the public sphere, and ends up triggering violence in the domestic environment, in the private sphere, as also observed in the ECU below.

*There was aggression once, without drinking it is a thing, totally different, but with drinking it is aggression, it is violent.* (*Int_08)

*I guess drinking makes him nervous, when he arrives angry, we have to hide all the knives, my daughter says I’m going to end up dying in his hands. I say I won’t let him, but I don’t sleep at night, I’m afraid of his violence.* (*Int_27)

The abusive use of alcohol and the potentiation of domestic violence have a direct impact on the SRH, especially with regard to reproductive planning, making women not want more children, and recognizing the deleterious effects on children, as seen in the interconnection between lexicons *going to* (phi= 0.36), *arrive* (phi= 0.28), *son* (phi= 0.22) and in the ECU below:

*My son has been asking for a little brother a lot, but I think I’ve been through so much trouble with his pregnancy that I don’t want it anymore. My husband used to drink a lot and was very violent, so I think, is this going to get better? Will he stop drinking? He says he wants another child, but is he going to change?* (*Int_08)

*My daughter was so afraid of her father that she would run screaming in fear. She would curl up in a corner and cover her ears when he arrived drunk and cursing.* (*Int_03)

Faced with the domestic violence experienced, rural women use mechanisms to cope with this situation. The lexicons *son* (phi= 0.22), *hide* (phi= 0.20), *talk* (phi= 0.08) and the ECU below illustrate this theme, which are the attitudes taken by women to live with domestic violence.

*Sometimes I’m out with my son and I see him drinking, I go home and take an anxiolytic to sleep. He starts drinking, and I pretend I don’t see it, I feel isolated. He gets home dizzy, I go out with my son, I stay in the square waiting until his dad falls asleep, the child feels it and is sad seeing his dad’s aggressiveness.* (*Int_08)

*Sometimes I ask him to talk, I tell him to discuss his problems and what am I doing wrong for him to be so aggressive and violent, but he won’t talk.* (*Int_17)

### Class 3 – The SRH of rural women in the family context: from standardization to the influence of family and domestic violence

Class 3 consisted of 97 ECU and 74 analyzable words, corresponding to 16% of the corpus. In this class, the words that typify it, such as *sexual* (phi 46), *violence* (phi 0.32), *man* (phi 0.31), *beat* (phi 0.30), *influence* (phi 0.29), *life* ( phi 0.29), *fight* (phi= 0.23), demonstrate a close connection with contents of the ECU that refer to the reflexes of being a rural woman in the SRH.

For these women, SRH seems to be based on sexual life, which needs to be a routine within the traditional family context of marriage. However, sexual practice is influenced by factors related to the woman’s chronological age, the length of the relationship and, mainly, the family and domestic violence experienced. This issue is signaled in the following lexicons; *sexual* (p hi 46), *influence* (phi= 0.29), *fight* (phi= 0.23), *relationship* (phi= 0.17), *discussion* (phi= 0.16), *before* (phi=0.08), *old* (phi=0.07).

*Our sex life used to be routine, it was normal. As a wife, I had that obligation: you lay down, have sex, turn to the other side and fall asleep.* (*Int_04)

*My sex life at the beginning of the relationship was much better, everything in life at the beginning is better, this menopause thing, I lost the desire, I used to have a lot of desire, now it’s gone. I’m getting old!* (*Int_11)

*So, we drink, then there was this time when he started talking loudly to me, I didn’t like it, it was already annoying me, every time we drink, he talks loudly and starts those fights, and sex is really bad, an obligation.* (*Int_04)

*This issue of violence in the relationship makes sex very boring, dull, but it’s my obligation.* (*Int_22)

**Figure 3:**
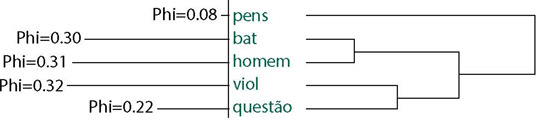
Ascending hierarchical classification of class 3 (PENS, beating, man, violence, issue)

By the contents of the ECU and the AHC, among the lexicons *beating* (phi= 0.30), *man* (phi= 0.31), *violence* (phi= 0.32), *issue* (phi= 0.22) (Figure 3), it is perceived that domestic violence is typified as physical aggression.

However, a small specific segment of participants presents a discourse of non-acceptance of this type of violence, prescribing attitudes that can break this situation and emphasizing the importance of respect in the relationship, according to the ECU below and the link between lexicons; *relationship* (phi= 0.21), *partner* (phi= 0.15), *exists* (phi= 0.15), *respect* (phi= 0.12).

*This issue of fighting between the couple, of men beating women, I think it should end, because there is no longer any respect.* (*Int_25)

*Every man who attacks a woman, she has to resort to the law that defends her, because the woman is very fragile before the man, she doesn’t have the strength he has.* (*Int_25)

## DISCUSSION

The SRs are developed from contexts to which the groups belong, as they are composed of contextualized beings^(22)^. The socioeconomic, demographic and SRH profile of participants showed that this is a context of women with low education, religious, in stable relationships, financially dependent on their partners and with an active sex life out of obligation. These characteristics were essential to understand the construction of identity and belonging functions, as well as their representations as rural women and the reflections on the SRH.

Social identities are shared by those who occupy the same positions and have common belongings and similarities^(13)^. In this context, the identity of rural women was built from socially imposed elements on the female segment, explained in motherhood as an inexorable destiny. Based on this logic, they understand that this is the only way they can find happiness. Following a different logic, investigations carried out with women in the urban context demonstrate that motherhood is not conceived as a natural destiny for all women, and the option of not having children is associated with other sources of personal satisfaction, such as higher levels of education, professional achievement, among others to which women direct their energies^(23, 24)^.

This representational line is rooted in the religious universe of participants, which is justified by the understanding that a social object is always apprehended as something associated with a group and its purpose^(14)^. For these women, the basic cultural assumption, anchored in religious belief and in the value given to motherhood as an identity, brought the new event to a circle in which it becomes potentially explainable. In this context, as advocated by the SRT, a symbolic relationship between the phenomenon and the subjects’ own lives is established^(25, 26)^.

This interpretation rests precisely on a cultural system, understood here as a pattern of meanings historically incorporated into a system of conceptions and practices. Motherhood brought to light the stereotype of the traditional woman, for whom building a family and having children are inherent characteristics of the female gender, while the opposite implies the denial of the woman’s nature, preventing her from being happy^(26)^.

Imbued with this conception, rural women develop the identity functions of the maternity and paternity groups of belonging based on gender inequalities. In our society, the boundaries of these roles are in a network of meanings located between the feminine and masculine^(23)^. Within these conceptions, there is a belief in the existence of the feminine, which is heir to philosophical, essentialist perspectives, quite resistant to change, especially in some groups such as those of religious and rural women^(27)^.

For these women, the traditional family stereotype is related to the representation of a dominant ideology, being a condition defined within a social structure of machismo, based on the social construction of identity and belonging of men and women^(27)^. Faced with this perspective, rural mothers assume themselves as the main care providers; for fathers, the understanding is that they have a secondary identity, objectified as helpers of the mothers in the care of children. This sexual division of tasks ends up legitimizing the ideology that caring for, giving affection and attention to children are exclusively female tasks, attributing to the male the responsibility for provision and education, however, in the sense of a punitive and coercive education. This organization of parenting translates gender inequalities elaborated through values, beliefs and individual expectations built from the cultural imaginary and social prescriptions^(27, 28)^.

In this sense, women’s SRs in the rural context seem to be permeated by the consensual universe and anchored in the imaginary of the patriarchal, traditional and nuclear family, crossed by the experience of intimate partner violence. Rural women recognize family and domestic violence, but in their speeches, the situation experienced is naturalized and normalized in their daily lives, and emerges an identity process of association between rural women and violence. Violence in the rural context is understood as intrinsic to women’s routine, as studies of this phenomenon in this social context are sometimes made invisible as a situation of gender violence, making it difficult to identify^(28, 29)^. In addition, given the geographical distance from urban areas, access to health, security and information services becomes more difficult in rural areas, and this isolation increases the chances of such women experiencing family and domestic violence^(30)^.

Women justify the aggressive behavior of their partners by the abusive use of alcohol, as if the locus of control of aggressiveness was solely external. In this representation, what determines and defines the path of one’s own existence comes from outside, with no possibility of control^(31)^. They present a justification dimension for the experience of this type of violence, minimizing the acts perpetrated by the partner^(25)^. There is a moral vision around the theme of family and domestic violence, which brings a social stigma that can lead to ostracism, rejection or submission^(15, 28)^. In addition, the educational level was evidenced as a factor that makes it difficult to break the circle of violence, as the low education increases the level of income deprivation, causing greater dependence of this woman on her partner^(32)^.

The SRs guide the way of jointly naming and defining the different aspects of daily reality, the way of interpreting these phenomena, making decisions and, eventually, taking a defensive position before them^(14)^.

Given this logic, rural women bring a practical dimension in the search for coexistence with this situation, making use of mechanisms to support living with aggressions, such as using anxiolytics, protecting their children, hiding objects that can be used as weapons and controlling the fertility, thereby preventing the conception of other children. However, they do not present attitudes that effectively break the cycle of violence. Studies reveal that one of the factors that lead women living in rural areas to present symptoms of depression is the lack of autonomy and control of their sexuality and fertility^(33, 34)^.

However, this representation was not unanimous. Another smaller segment of younger participants with higher education and who work outside the home prescribed socially acceptable behaviors they would have when experiencing family and domestic violence, such as breaking up with the partner and reporting to competent bodies. With this, they demonstrate the conventional and prescriptive nature of the SRs that model objects, people or events, according to the language, time and culture of a given group^(14)^. As this is a segment of women from another generation who also live in environments of the public sphere, they seem to be in a phase of transition and overcoming some values of patriarchy, which are transformed in parallel with other social changes, such as the woman’s identity function.

The representation of being a woman in the rural context permeated by family and domestic violence also brings an affective dimension that is reflected in the SRH, mainly in their sexual practice, reproductive planning and family dynamics in relation to the upbringing and education of children. Sexual and reproductive health seem to be anchored in the sexual relationship designed as a female obligation, standardized, without showing sexual desire and pleasure. The sexual experience is based on the patriarchal logic that attributed to the man the power to become the owner of the female body and allocates the sexual experiences always aimed at serving him, constituting a behavior to maintain the marriage^(27)^.

The group of belonging in this study, predominantly of Catholic women, sees marriage as a fundamental sacrament, with prescriptions of feminine virtues necessary for the formation of the traditional family, such as marriage, chastity and obedience to men. The normalization of attitudes is one of the characteristics of SRs, which are endowed with conventional and prescriptive power over reality and the social environment^(14, 28)^.

**Figure 4:**
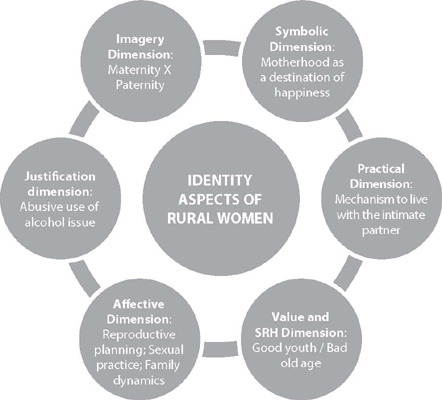
Representation field

The age factor was also identified as a discouraging aspect of sexual practice, interfering with their SRH. Historically created symbols between youth and old age are developed in the public sphere, and these intersubjective productions influence everyday life, especially for women^(35, 36)^. This makes these representations (shared in collective scenarios) progressively maintain the SRs of rural women, which brings the evaluative dimension of a good sexual practice in youth and bad in old age. Faced with these perspectives, the SRH experience seems to restrict or even annul the possibility for women to enjoy their sexual and reproductive rights freely without coercion and violence.

In short, the rural woman affected by the fear of experiencing situations of embarrassment and difficulty in raising children because of family and domestic violence chooses not to have more children as a defense mechanism, even though it does not reflect her real desire. Most of the time, the social role of rural women is restricted to the home environment, supported by relationships of subordination and obedience, in which the man is seen as the reference in the family unit. Gender stereotypes already established and predominant in a patriarchal society are reaffirmed in this scenario^(27)^.

### Study Limitations

The limitation is the fact that this study was conducted with women residing in the rural area of only one municipality in the countryside of the state of Minas Gerais. Participants represent their social objects from their practical experiences in everyday life and bring peculiar aspects of the studied region. However, the results of this study can contribute to recognize the specificities of this group and stimulate the reflection on other studies performed in the rural context.

### Contributions to the Area of Nursing, Health or Politics

Gender inequalities and the singularities of minority groups such as rural women demonstrate the challenges to promote a liberating SRH that involves the transformation of health practices based on the logical principle of prescription and control to the principle of ethics and freedom. The scientific knowledge underlying health actions must guarantee reproductive and sexual rights, breaking actions that end up repressing and perpetuating conservative morals, reinforcing the heteronomy to which rural women are subjected, as evidenced in this article. Nursing has the potential to perform these liberating actions in the SRH of rural women, given their presence in the rural context through Primary Health Care with a protagonist role in actions of prevention and promotion of women’s health.

## FINAL CONSIDERATIONS

In this study, it was possible to understand the identity components articulated in the field of SRs of being a rural woman, as elaborated from their daily lives, as well as in other elements of a symbolic, normative, evaluative and affective nature that mobilized subjectivities related to their social belonging.

The woman’s identity in the rural context is anchored in gender inequality, which constructs her as a dedicated, abdicated wife and family supporter, naturalizing family and domestic violence, which ends up reflecting negatively on her SRH. Rural women find themselves inserted in a patriarchal society, subject to the norms and prescriptions of this paradigm in which the locus of control of their lives and their bodies lies in the other, whether in religiosity or in the partner.

It is urgent to increase attention on SRH in an egalitarian and liberating way in order to minimize the consequences of machismo and conservatism that end up imprisoning and standardizing culturally imposed obligations on rural women, taking away their sexual and reproductive rights.

## References

[1] Leite SP (2020). Ruralidade, enfoque territorial e políticas públicas diferenciadas para o desenvolvimento rural brasileiro: uma agenda perdida?. Estud Soc Agric.

[2] Gonçalves H, Tomasi E, Tovo-Rodrigues L, Bielemann RM, Machado AKF, Ruivo ACO (2018). Population-based study in a rural area. Rev Saúde Pública.

[3] Bousquat A, Fausto MCR, Almeida PF, Lima JG, Seidl H, Sousa ABL (2022). Different remote realities: health and the use of territory in Brazilian rural municipalities. Rev Saude Publica.

[4] Arruda NM, Maia AG, Alves LC (2018). Desigualdade no acesso à saúde entre as áreas urbanas e rurais do Brasil: uma decomposição de fatores entre 1998 a 2008. Cad Saúde Pública.

[5] Miranda SVC, Duraes PS, Vasconcellos LCF (2020). A visão do homem trabalhador rural norte-mineiro sobre o cuidado em saúde no contexto da atenção primária à saúde. Cien Saúde Coletiva.

[6] Bortolotto CC, Loret de Mola C, Tovo-Rodrigues L (2018). Qualidade de vida em adultos de zona rural no Sul do Brasil: estudo de base populacional. Rev Saúde Pública.

[7] Maraschin MS, Souza EAD, Caldeira S, Gouvêa LAVND, Tonini NS (2019). Perfil sociodemográfico e econômico de mulheres trabalhadoras rurais. Rev Nurs.

[8] Ministério da Saúde (BR) (2013). Política Nacional de Saúde Integral das Populações do Campo e da Floresta [Internet].

[9] Delgado VC, Bedmar VL (2020). Women’s Well-Being and Rural Development in Depopulated Spain. Res Public Health.

[10] Baker KK, Padhi B, Torondel B, Das P, Dutta A, Sahoo KC (2017). From menarche to menopause: a population-based assessment of water, sanitation, and hygiene risk factors for reproductive tract infection symptoms over life stages in rural girls and women in India. PLoS One.

[11] Arboit J, Costa MC, Silva EB, Colomé ICS, Prestes M (2018). Violência doméstica contra mulheres rurais: práticas de cuidado desenvolvidas por agentes comunitários de saúde. Saúde Soc.

[12] Costa MC, Silva EB, Soares JSF, Borth LC, Honnef F (2017). Rural women and violence situation: access and accessibility limits to the healthcare network. Rev Gaúcha Enferm.

[13] Arruda A (2002). Teoria das representações sociais e teorias de gênero. Cad Pesq.

[14] Moscovici S (2017). Representações sociais: investigações em psicologia social.

[15] Jodelet D, Jodelet D (2001). As representações sociais.

[16] Jovchelovitch S, Almeida AMO, Santos MFS, Trindade ZA (2014). Teoria das representações sociais - 50 anos [Internet].

[17] Instituto Brasileiro de Geografia e Estatística (IBGE) (2021). Vamos conhecer o Brasil [Internet].

[18] Minayo MCS (2017). Amostragem e saturação em pesquisa qualitativa: consensos e controvérsias. Rev Pesqui Qualit.

[19] Ferreira MCG, Tura LFR, Silva RC, Ferreira MA (2017). Social representations of older adults regarding quality of life. Rev Bras Enferm.

[20] Oliveira LAF, Oliveira AL, Ferrera MA (2021). Nurses' training and teaching-learning strategies on the theme of spirituality. Esc Anna Nery.

[21] Apostolidis T (2006). Representations sociales et triangulation: une application en psychologie sociale de la santé. Psic Teor Pesq.

[22] Arruda A (2009). Teoria das representações sociais e ciências sociais: trânsito e atravessamentos. Soc Estado.

[23] Machado JSA, Penna CMM, Caleiro RCL (2019). Cinderela de sapatinho quebrado: maternidade, não maternidade e maternagem nas histórias contadas pelas mulheres. Saúde Debate.

[24] Crisostomo KN, Grossi FRS, Souza RS (2019). As Representações Sociais da Maternidade para Mães de Filhos/as com Deficiência. Rev Psicol Saúde.

[25] Nóbrega VKM, Pessoa JM, Nascimento EGC, Miranda FAN (2019). Resignation, violence and filing complaint: social representations of the male aggressor from the perspective of the female victim of aggression. Ciênc Saúde Coletiva.

[26] Cunha ACB, Eroles NMS, Resende LM (2020). Tornar-se mãe: alto nível de estresse na gravidez e maternidade após o nascimento. Inter Psicol.

[27] Rodrigues HF (2017). O pai odioso: o feminino na família patriarcal. Rev Estud Linguíst Literários.

[28] Acosta DF, Gomes VLO, Oliveira DC, Marques SC, Fonseca AD (2018). Representações sociais de enfermeiras acerca da violência doméstica contra a mulher: estudo com abordagem estrutural. Rev Gaúcha Enferm.

[29] Zakar R, Zakar MZ, Abbas S (2016). Domestic violence against rural women in Pakistan: an issue of health and human rights. J Fam Viol.

[30] Gokler ME, Arslantas D, Unsal A (2014). Prevalence of domestic violence and associated factors among married women in a semi-rural area of western Turkey. Pak J Med Sci.

[31] Rouquete ML, Moreira ASP, Oliveira DC (2000). Estudos interdisciplinares de representação social.

[32] Dillon G, Hussain R, Loxton D, Khan A (2016). Rurality and Self-Reported Health in Women with a History of Intimate Partner Violence. PLoS ONE.

[33] Parreira BDM, Goulart BF, Ruiz MT, Silva SR, Gomes-Sponholz FA (2017). Depression symptoms in rural women: sociodemographic, economic, behavioral, and reproductive factors. Acta Paul Enferm.

[34] Alano A, Hanson L (2018). Women's perception about contraceptive use benefits towards empowerment: A phenomenological study in Southern Ethiopia. PLoS One.

[35] Oliveira EL, Neves ALM, Silva IR (2018). Sentidos de sexualidade entre mulheres idosas: relações de gênero, ideologias mecanicistas e subversão. Psicol Soc.

[36] Souza CL, Gomes VS, Silva RL, Santos ES, Alves JP, Santos NR (2019). Aging, sexuality and nursing care: the elderly woman’s look. Rev Bras Enferm.

